# l-Arginine Grafted Poly(Glycerol Sebacate) Materials: An Antimicrobial Material for Wound Dressing

**DOI:** 10.3390/polym12071457

**Published:** 2020-06-29

**Authors:** Chia-Chun Wang, Ting-Yu Shih, Yi-Ting Hsieh, Jie-Len Huang, Jane Wang

**Affiliations:** 1 Department of Chemical Engineering, National Tsing Hua University, Hsinchu 30013, Taiwan; ch19860826@hotmail.com; 2 Laboratories of Materials and Chemical Research, Industrial Technology Research Institute, Hsinchu 31057, Taiwan; tingyu@itri.org.tw (T.-Y.S.); Michelle_Hsieh@itri.org.tw (Y.-T.H.); jielenhuang@itri.org.tw (J.-L.H.); 3 R&D Center for Membrane Technology, Chung Yuan Christian University, Taoyuan 32023, Taiwan

**Keywords:** l-arginine, poly(glycerol sebacate), antimicrobial function, wound dressing

## Abstract

This study focuses on the development and evaluation of a novel wound dressing material. l-arginine grafted poly(glycerol sebacate) materials (PGS-g-Arg) are developed by chemical conjugation of l-arginine on poly(glycerol sebacate) chains and the mechanical property, water vapor transmission rate, antimicrobial functions and biocompatibility are investigated. At various l-arginine grafting ratio, the mechanical properties are tunable. It was found that between 7–13% l-arginine grafting ratios, the tensile strengths of PGS-g-Arg were similar to that of natural skin. These materials are shown with a low water vapor transmission rate, 6.1 to 10.3 g/m^2^/h, which may form a barrier and assist in the closure of wounds. Inhibition in the growth of *Pseudomonas aeruginosa* and *Staphylococcus aureus* was observed on PGS-g-Arg, and a series of experiments were conducted to confirm its biocompatibility. In summary, l -arginine grafted poly(glycerol sebacate) may offer a novel option for wound dressing.

## 1. Introduction

Wound dressings play an important part in the treatment of injured skin. They usually cover external wounds to maintain the hydration of skin and help with wound healing. Among various materials for wound dressing, silicone-based materials, especially silicone gel sheetings (SGS), are clinically proven to significantly prevent scar formation and assist with scar treatment. In theory, scars are formed when a large amount of water vapor evaporates from wounds, leading to keratinocytes dehydration to stimulate fibroblast to form fibrosis tissues [1]. Silicone patches are soft materials, which may conform to skin and exhibit a low water vapor transmission rate, forming a barrier to decrease water vapor evaporation from wounds [2]. More and more studies indicate that the key functions of silicone-related products are likely to assist in the closure and hydration of wound sites [1,3]. In addition, since wound treatment usually takes a period of time, it is possible that bacterial infection will occur during the repair process, which increases the risk of scar formation and subsequently prolongs the treatment cycle. However, as silicone-based wound dressings lack appropriate antimicrobial functions, the use of such wound dressing may raise the risks of second infection. In order to avoid the problems mentioned, onion extracts are included in some of the products for scar treatment. However, allergic reactions and inflammations were observed in some cases when using such products on the wound skin [4]. On the other hand, silver nanoparticles are known as additional antibacterial ingredients in some products, yet the biocompatibility and toxicity of silver nanoparticles are divergent in many studies [5,6]. Overall, these conditions raised questions to the stability and biocompatibility of such additives.

Based on the above descriptions, wound dressings that are antimicrobial yet biocompatible are needed. In this study, a novel material, l-arginine grafted poly(glycerol sebacate) (PGS-g-Arg), is developed for the application of wound dressing and is evaluated for antimicrobial efficacy. Poly(glycerol sebacate) (PGS) is a biocompatible material first developed by Wang et al. in 2002 [7]. The Young’s modulus was tunable by altering monomer ratios and curing parameters, 0.3 to 1.2 MPa, which was similar to skin tissue [8]. Recently, PGS-based products have begun hitting the market, for example, Regenerez^®^, and have demonstrated high applicability in medical applications. Meanwhile, according to the studies of Pérez et al. [9], Morán et al. [10], and Pinazo et al. [11], l-arginine-based surfactants exhibit antimicrobial functions. It is generally believed that the positively charged l-arginine may become adsorbed onto the negatively charged bacterial membranes through electrostatic attractions. As ion channels are formed, the fluidity and permeability of the cell membrane change, leading to the inhibition of intracellular carbon metabolism and damaging bacteria. In addition, l-arginine is a naturally occurring amino acid in human body and has been used in many cosmetic products. l-arginine is also a food additive permitted by the Food and Drug Administration (FDA) [12]. Therefore, the combination of PGS polymer and l-arginine may pose a possible solution for wound dressings. In this study, in the attempts to apply these ideas in wound dressings, l-arginine was grafted on PGS polymers (PGS-g-Arg) by chemical modification, and the polymer networks were obtained by further chemically crosslinking reaction (c(PGS-g-Arg)). Through the analyses of the mechanical properties, swelling ratio, water vapor transmission rate, antimicrobial function, and cytocompatibility, the characteristics of these materials were investigated.

## 2. Materials and Methods

### 2.1. Materials

All materials were purchased from Sigma-Aldrich (St. Louis, MO, USA), and used as received unless otherwise specified.

### 2.2. Preparation of Poly(Glycerol Sebacate) and l-arginine Modified Poly(Glycerol Sebacate) Prepolymer

PGS-g-Arg prepolymer was synthesized by two steps. First, 17 h melted sebacic acid and glycerol with equal molar amount underwent polycondensation reaction under the atmospheric pressure of 1.5 to 3 torr at 130 °C for 8 h to form PGS prepolymer. Second, PGS prepolymer dissolved l-arginine with the amounts of 15 mol%, 30 mol%, and 50 mol% of hydroxyl groups on PGS prepolymer under nitrogen purge at 130 °C for 2.5 h. The solution was chemically modified under the atmospheric pressure of 3 to 8 torr at 110 °C for 24 h. The mixture was then dissolved in methanol and purified by dialysis (BIOTECH dialysis membrane Spectra/Pro3, MWCO: 3500) in de-ionized water (DIW) for 2 days and in methanol for 8 h. Finally, the purified solution was dried by rotary evaporation to get PGS-g-Arg prepolymer and the grafting ratio of l-arginine on PGS prepolymer backbone was characterized by Nuclear Magnetic Resonance (NMR) spectrometer (Varian INOVA500, USA). The chemical composition was determined by the signal at 1.4, 1.6, and 2.2–2.4 ppm for sebacic acid, and at 3.5–4.2 ppm and 5.1–5.3 ppm for glycerol, and at 1.7, 1.9 and 3.2 ppm for l-arginine, and at 4.3 ppm for l-arginine-substituted glycerol. The molecular weight of prepolymers were measured by gel permeation chromatography (GPC) on an Agilent instrument with tetrahydrofuran as eluent at a flow rate of 1.0 mL/min, and a polystyrene standard was used for calibration.

### 2.3. Preparation of Poly(Glycerol Sebacate) and L-arginine Modified Poly(Glycerol Sebacate) Crosslinking Films

Square flat glass substrates (11 cm × 11 cm) were first coated with 4% carboxymethyl cellulose solution and dried at 50 °C for 4 h as a released film. Next, ethanol solution containing PGS-g-Arg prepolymer was gently poured on the glass substrates and the solvent was removed by drying at 80 °C for 24 h in air circulation oven plus 1 h in vacuum oven. The prepolymer was crosslinked under vacuum (0.8 to 1 torr) at 150 °C for 72 h plus 160 °C for 24 h for the further crosslinking. Final, the crosslinked PGS (c(PGS)) or PGS-g-Arg films (c(PGS-g-Arg)) were obtained after soaking in 70 °C hot water overnight and drying in the oven at 70 °C. Both structures of crosslinked PGS and PGS-g-Arg films were characterized by ATR (Thermo Fisher Scientific, Waltham, USA, Nocolet iS50 FT-IR spectrometer) and compared against each other.

### 2.4. Mechanical Property Test

The c(PGS-g-Arg) films were punched to the size of 30 mm × 3 mm (*n* = 5) and tested on an TA-ElectroForce^®^ 3200 Series III system (Thermal Analysis, USA) with a 225 N load cell. Specimens were extended at a rate of 0.05 mm/min until break. The data of ultimate tensile strength and elongation at break were collected, and Young’s modulus (MPa) was calculated from the slope of the first 10% of the stress–strain curve.

### 2.5. Swelling Ratio Test

Swelling by hydration of c(PGS-g-Arg) was measured through the mass differences after soaking crosslinked films in DIW at 23 °C for 24 h. American Society for Testing and Materials (ASTM) D570 was used as a referenced protocol [13]. The c(PGS-g-Arg) was punched into circular films with 16 mm diameter (*n* = 3). Each specimen was then dried in an oven at 50 °C for 24 h and cooled in a desiccator to reach the mass equilibrium (W0). After weighing the dried specimens, DIW was added to each specimen and the specimens were entirely immersed. At the end of 24 h, each specimen was removed from water and the surface was wiped dry. The mass of wet specimens was then weighed immediately (W1). The swelling ratio of each specimen was calculated by dividing the differences of wet specimen and dried specimen with dried specimen ((W1 − W0)/W0 × 100%).

### 2.6. Water Vapor Transmission Rate (WVTR) Test

In this test, three commercial silicone dressings, i.e., Cica-Care^®^ (Smith and Nephew, Watford, UK), SavDerm^®^ (Oriental Resources Development Limited, New Taipei City, Taiwan) and Rystora^®^ (Fortune Medical, New Taipei City, Taiwan), were purchased and used for comparative purposes.

#### 2.6.1. Vial Method

Tested specimens, including commercial products, were punched into circular films with 16 mm diameter (*n* = 3). As the suggestion in ASTM E96 [14], the water level should be 19 ± 6 mm from the specimen. Therefore, 20 mm glass vials were filled with 16 mL distilled water. Each specimen was placed on the center of the vial opening with hollow septa (10 mm diameter), and the tested vials were secured with aluminum ring by a crimper hand sealing tool. The diameter of the vial opening was 10 mm, which meant the area of water vapor transmission (WVT) was 7.9 × 10^−5^ m^2^. Tested vials were placed at the temperature of 37 ± 2 °C and relative humidity of 25 ± 5% for 48 h. The mass change of water in each tested vial was weighted, and the water vapor transmission rate (WVTR) of each specimen was calculated and expressed in mass units (g) per area (m^2^) per hour. Vials without covered materials were used as a 0% occlusive control of water.

#### 2.6.2. Mocon Method

Mocon is recognized as one of standard instruments for measuring WVTR; thus, one of the commercial products and one of c(PGS-g-Arg) films with similar thickness were examined. Tested films with 5 × 5 cm (*n* = 2) were placed on the diffusion cells (Mocon PERMATARN W 3/61), and the water vapor were blown 1 h for system equilibrium. After reaching 90% relative humidity and 40 °C, the WVTR of each tested films was recorded at each time point until 18 h.

### 2.7. Antimicrobial Test

The antimicrobial tests of c(PGS-g-Arg) films were examined according to Japanese Industrial Standards (JIS) Z2801: test for antimicrobial function of plastics [15]. The nutrient broth inoculated with bacterial strains for the tests, including *Staphylococcus aureus* (ATCC^®^6538) and *Pseudomonas aeruginosa* (ATCC^®^9027). Cultured bacteria were prepared and diluted to the concentration of 2.5 × 10^5^ CFU/mL as the test inoculums. 0.4 mL of each inoculum was dropped onto each sterilized tested film (5 × 5 cm, *n* = 2) and covered with a clean polyethylene film (4 × 4 cm), then the test inoculum spread evenly over the film surface in a petri dish. After 24 h incubation at 35 °C, each tested film was flushed with 10 mL SCDLP broth to wash out the test bacteria. The cell count of bacteria was performed by taking 1 mL of the washing solution and 9 mL PBS buffer for serial dilution and the appropriate diluted solution was plated on counting agar (Difco) to incubate for another 24 h. After incubation, bacterial colonies were counted and recorded as S. The clean PE film was used as s control group (C). Therefore, the value of antimicrobial activity (R, microbial log reduction) and antimicrobial efficacy (%) were calculated by the following equations:

The value of antimicrobial activity:

*R* = log *C* − log *S*

Antimicrobial Efficacy% = (*C* − *S*)/*C* × 100%

where, *C* is the average of numbers of counting bacteria from control and *S* is the average of numbers of counting bacteria from tested film.

### 2.8. In-Vitro Cytocompatibility

To evaluate the cytocompatibility of the films, the tests on agar diffusion from International Organization for Standardization (ISO) 10993-5 [16] and ASTM F895-11 [17] were used as referenced protocols. 2.5 × 10^5^ cell/well L929 cell lines (The National Collection of Type Culture (NCTC) clone 929, mouse subcutaneous connective tissue) were seeded in 6 well culture plates with 2 mL growth medium (90% Minimum Essential Media (Gibco) and 10% fetal bovine serum (Gibco) and supplemented with 1% penicillin-streptomycin (Gibco)) and cultured at 37 °C and 5% CO_2_. After 48 h, the culture medium was replaced with the equal amount of medium containing 1.5% agar (Nobel agar, BD Difco^TM^) and each tested film, punched into the disks with 10 mm diameter (*n* = 3), and carefully placed on the center of solidified agar layer. After incubation for 24 h, each specimen was removed and each culture well was dropped 2 mL culture medium containing 0.01% neutral red solution for 1 h. The reactivity grades of zone and morphological grades of cytotoxicity of tested specimens were observed and evaluated by microscope (Nicon, ECLIPSE-TS100). Rubber and Teflon films were used as positive and negative control, respectively.

## 3. Results and Discussion

### 3.1. Structural Analysis of Prepared Polymers

**Figure 1:**
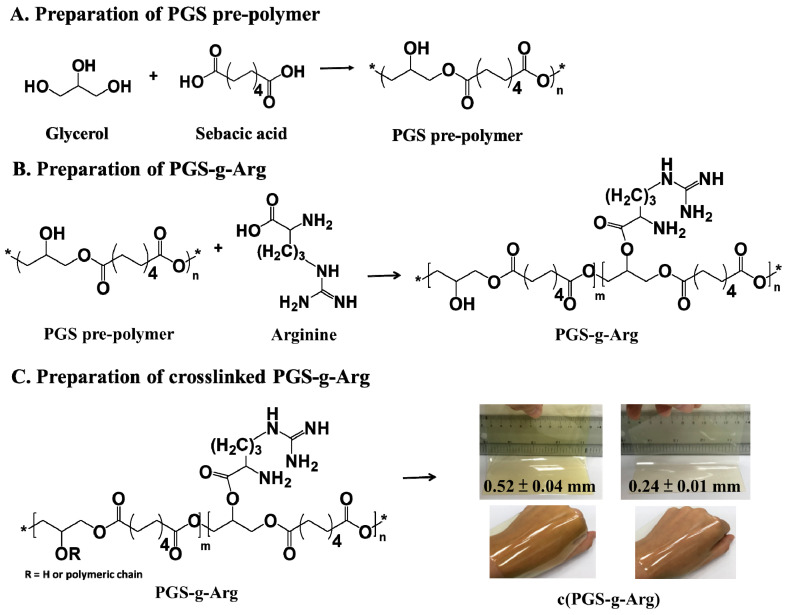
The preparation schemes of (**A**) poly(glycerol sebacate) (PGS) prepolymer, (**B**) PGS-g-Arg polymer, and (**C**) PGS-g-Arg crosslinked films (c(PGS-g-Arg)).

**Figure 2:**
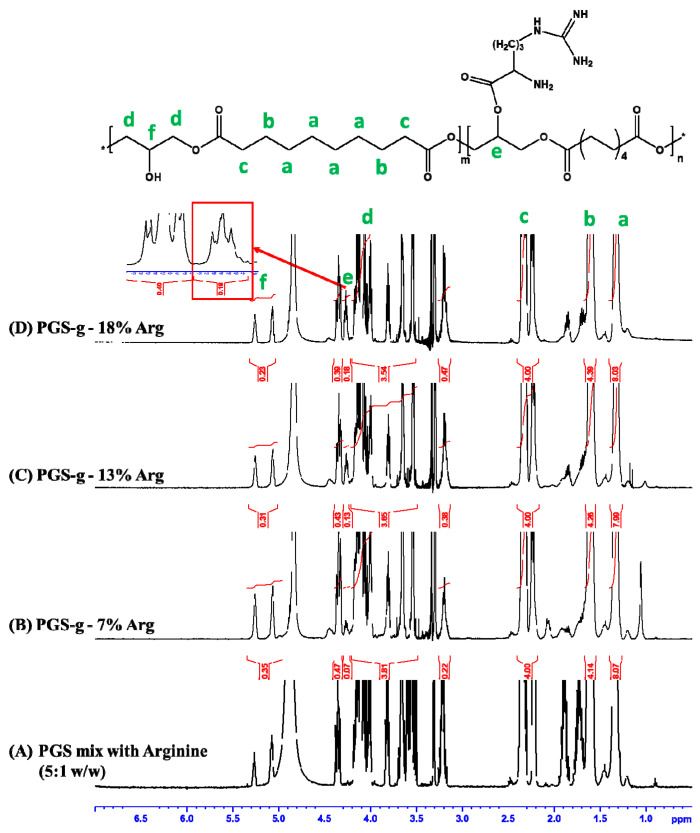
The relative positions and structural analyses of (**A**) PGS prepolymer mix with l-arginine (5/1 *w*/*w*) and PGS-g-Arg prepolymers with the grafting content of (**B**) 7%, (**C**) 13%, and (**D**) 18% l-arginine, respectively on ^1^H-NMR spectra (d-solvent: CD_3_OD).

**Figure 3:**
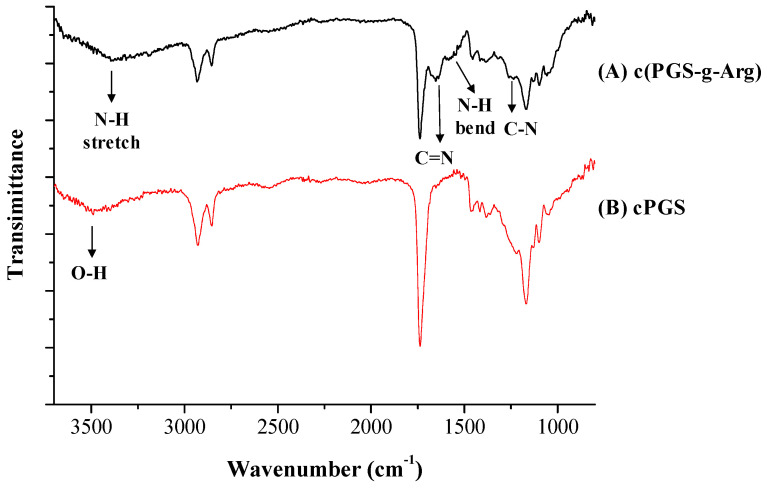
Attenuated Total Reflection-Fourier transform infrared (ATR-FTIR) spectra of (**A**) crosslinked PGS-g-Arg film (c(PGS-g-Arg)) and (**B**) crosslinked PGS film (c(PGS)).

The preparation procedures of PGS-g-Arg prepolymers are shown in Figure 1A,B. PGS-g-Arg prepolymer was prepared by reacting PGS prepolymer with L-arginine, and was characterized by proton nuclear magnetic resonance (^1^H-NMR) spectrometer. The corresponding chemical shifts of proton signals on the prepolymers were identified and labeled in Figure 2. The mixture of PGS prepolymer and l-arginine before reactions was revealed in Figure 2A. Comparing between Figure 2A–D, it was noticed that a signal at 4.3 ppm was detected in Figure 2B–D. Since the l-arginine compounds grafted on PGS prepolymers could be considered as electron-donating groups, the shielding effect of protons from the tri-substituted glycerol on the prepolymers (5.1 to 5.3 ppm) was increased leading to the upfield shift to 4.3 ppm. This confirmed that the carboxyl group on l-arginine compounds successfully replaced the hydroxyl groups on PGS prepolymers after synthesis. Therefore, the integral values at 4.3 ppm and the integral value of methylene groups of PGS prepolymer between 2.2 to 2.4 ppm were used to calculate the grafting ratio of l-arginine. In this study, three different grafting ratios of L-arginine on PGS prepolymers were obtained, which were 7%, 13% and 18%, respectively. PGS-g-Arg prepolymers were then dissolved in methanol and casted on the mold for polycondensation reaction. Over the course of the reaction, the residual hydroxyl groups reacted until the crosslinked films (c(PGS-g-Arg)) were obtained, and the transparent and uniform films are shown in Figure 1C. The dimensions of each c(PGS-g-Arg) film were compiled in Table 1. Through altering the solid contents of PGS-g-Arg solution, the thickness of the film is adjustable. The structural analyses of crosslinked films by ATR are shown in Figure 3A. Typical absorption peaks of hydroxyl (3470–3490 cm^−1^), ester (1170 cm^−1^ and 1740 cm^−1^), and alkyl groups (2850–2930 cm^−1^) in the c(PGS-g-Arg) films were shown, as was observed in crosslinked PGS films (c(PGS)) (Figure 3B). Besides that, absorption peaks of the amine (3400 cm^−1^, 1570 cm^−1^ and 1260 cm^−1^) and imine groups (1650 cm^−1^) were also shown in Figure 3A, which were associated with l-arginine groups. From the characterization of ^1^H-NMR and ATR-FTIR spectra, PGS-g-Arg polymers were successfully synthesized in this study.

### 3.2. Analyses of Mechanical Property and Swelling Ratio of Crosslinking Films

**Figure 4:**
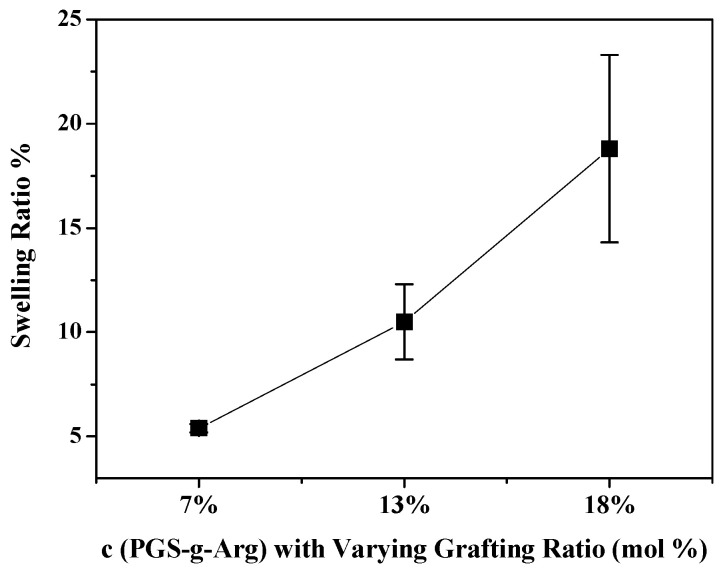
Comparison of swelling ratio of c(PGS-g-Arg) films with varying grafting ratios (*n* = 3).

Considering that the intended uses of these novel materials are as wound dressings, the elasticity of the materials should mimic that of natural skins, of which the Young’s modulus was between 0.5 to 1.95 MPa [8]. Therefore, the tensile tests on the c(PGS-g-Arg) films were investigated and are shown in Table 2. The Young’s modulus of crosslinked films between 7% and 13% grafting ratio of l-arginine were comparable with natural skin. It was noticed that the Young’s modulus of the crosslinked polymers were between 0.37 MPa to 1.79 MPa under varying grafting ratios of l-arginine. Upon scrutiny, the Young’s modulus and ultimate tensile strength (UTS) of the c(PGS-g-Arg) films were decreased with the increasing grafting ratios of l-arginine while the elongation at breaks were reversed. This was likely attributed to varying grafting ratios of l-arginine on polymers, leading to the differences of degree of crosslinking density. The higher l-arginine contents, which resulted in lower hydroxyl group contents, would limit the formation of crosslinked chains, leading to the relatively soft tensile modulus of c(PGS-g-Arg) films. This also explained why the swelling ratio of c(PGS-g-Arg) films were decreased with the increasing grafting ratios of l-arginine. As shown in Figure 4, the swelling ratios of 7%, 13%, and 18% c(PGS-g-Arg) films were 5.4%, 10.5%, and 18.8%, respectively. Therefore, the grafting ratio of polymers may be controlled properly to meet the applicability.

### 3.3. Water Vapor Transmission Rate of Crosslinking Films and Commercial Products

**Figure 5:**
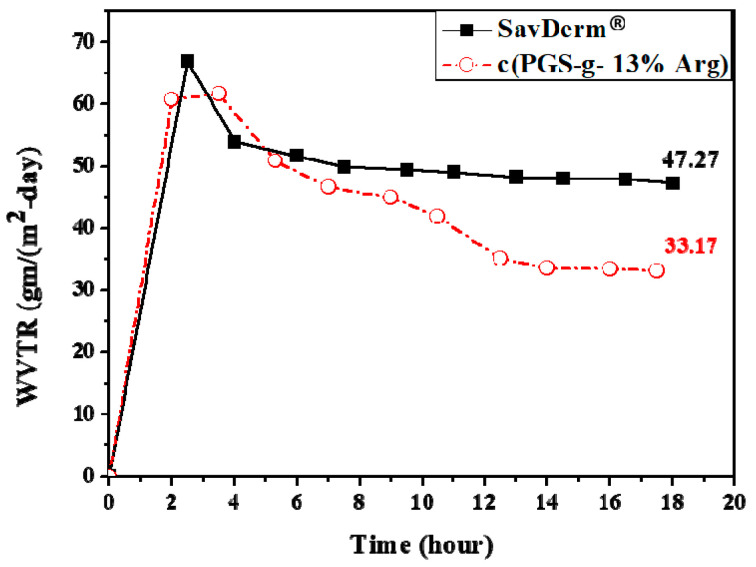
Comparison of the WVTRs of c(PGS-g- 13% Arg) with SavDerm^®^ by Mocon method (*n* = 2).

According to the studies of Lamke et al. and Tandara et al., the average value of WVTR is 15 g/m^2^/h in healthy skin, while the value of WVTR in wounded skin, especially burnt skin, is much higher than that [18,19]. Therefore, in order to maintain the physiological repair functions of skin, it is crucial that the dressing must have the function to form a barrier and decrease water vapor evaporation from wounds. Two typical methods, the standard vial method and the Mocon instrument, were used to determine the water vapor transmission rate (WVTR) of materials in many studies. In this study, these two methods were used to determine the water vapor transmission rate (WVTR) of the c(PGS-g-Arg) films and commercial products. The standard vial method was based on ASTM E96 (standard test methods for water vapor transmission of materials). Within 48 h of testing, all samples reached the equilibrium state and the results are shown in Table 3. The value of WVTR of 7%, 13%, and 18% c(PGS-g-Arg) films with the thickness from 0.46 to 0.50 mm were 6.1, 7.7, and 8.3 g/m^2^/h, respectively. For c(PGS-g-Arg) films with similar thicknesses, the effects of varying grafting ratios of l-arginine toward the properties of WVTR were very similar. On the other hand, the effects of film thickness toward the properties of WVTR were clearly observed. Under the same grafting ratio of l-arginine, 7% grafted c(PGS-g-Arg) film, the WVTR at the thickness of 0.24 mm film was 10.3 g/m^2^/h. This result was quite different when compared to the thickness of 0.46 mm film, which was 6.1 g/m^2^/h. It was also found that the WVTR of Rystora^®^ with 0.3 mm thickness was 12.2 g/m^2^/h, which was higher than other commercial products with thicker films, Cica-Care^®^ (7.2 g/m^2^/h) and SavDerm^®^ (7.8 g/m^2^/h). According to the results of the vial method, the WVTR of these materials were highly correlated to the thickness of the film, between 0.2 to 0.5 mm. c(PGS-g-13%Arg) film and SavDerm^®^, which corroborated with the WVTR obtained through the vial method, were selected to test in Mocon method and the results are shown in Figure 5. After 12 h, two tested samples reached equilibrium and in the end of test, the value of WVTR of c(PGS-g-13% Arg) was 33.17 gm/m^2^-day, which outperformed SavDerm^®^ in water-sustained ability, which was up to 47.27 gm/m^2^-day. Overall, both methods proved that the WVTR of the developed materials, c(PGS-g-Arg) films are comparable to the commercial products, which indicates that c(PGS-g-Arg) are capable of assisting with the closure and hydration of wounds.

### 3.4. Determination of Antimicrobial Function

**Figure 6:**
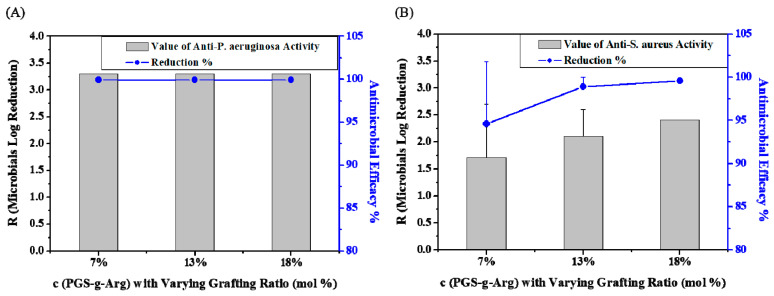
The capability of (**A**) anti-*Pseudomonas aeruginosa* and (**B**) anti-*Staphylococcus aureus* of c(PGS-g-Arg) films (*n* = 2).

In the 1980s, the idea that positively charged material has unique physiological effects to reduce the colony formation of bacteria was first proposed, and has become a key concept in the design of antimicrobial materials in recent decades. It is worth noting that the proper density of positive charges is one of the key factors for successful antimicrobial activity [11,20,21,22,23,24]. *Pseudomonas aeruginosa* and *Staphylococcus aureus* are two of the most common microbes that commonly lead to infections on wounds, and are also used to examine the antimicrobial function of materials in many studies [25,26,27]. Therefore, c(PGS-g-Arg) films with varying grafting ratios of l-arginine were tested with these two microbes to evaluate the antimicrobial function. After 24 h of incubation, there were almost no *Pseudomonas aeruginosa* remaining on the counting agar. As suggested in JIS 2801, the bacterial colonies were recorded as <1 and the results are shown in Figure 6A. It was found that the log reduction of *Pseudomonas aeruginosa* at c(PGS-g-Arg) films with a varying grafting ratio of l-arginine were 3.3, which meant that the efficacy of anti-*Pseudomonas aeruginosa* was above 99.9%. According to the JIS 2801 standard, the antimicrobial material is determined when the log reduction of microbial is ≥2 [28]. Therefore, the performances of c(PGS-g-Arg) films on anti-*Pseudomonas aeruginosa* were quite efficient as the grafting ratio of l-arginine was above 7%. On the other hand, as shown in Figure 6B, the effect of anti-*Staphylococcus aureus* was obviously dependeny on the grafting ratio of l-arginine. The log reductions of *Staphylococcus aureus* at 7%, 13%, and 18% c(PGS-g-Arg) films were 1.7, 2.1, and 2.4, respectively. These results indicated that the antimicrobial activity may be linked to the adequate degree of charged density presented on materials. Therefore, in order to inhibit both *Pseudomonas aeruginosa* and *Staphylococcus aureus*, the proper grafting ratio of l-arginine on c(PGS-g-Arg) should be ≥13%.

### 3.5. Cytocompatibility Evaluation

**Figure 7:**
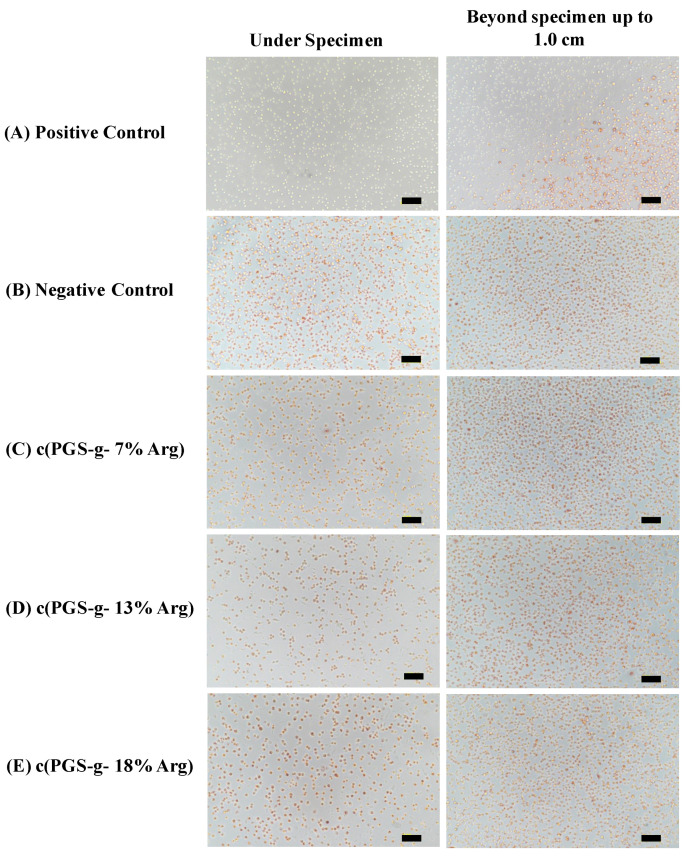
Optical observation of L929 cell lines via neutral red staining (scale bar 100 μm).

Considering the potential application of developed material l-arginine modified poly(glycerol sebacate) polymer might be as wound dressings, the in-vitro cytocompatibility of these novel materials were evaluated to meet the essential criteria of the uses for medical devices. After incubation with L929 cell for 24 h, the specimens were removed from the culture wells and the viability of cells were observed after neutral red staining. Neutral red staining is a typical method to assess the cell viability by the specific lysosomal capacity of cells for ingesting the dye [29]. If cells were injured or dead, the color of red would not be revealed, which could provide the obviously visual observation for grading the cytotoxicity (Figure 7). According to ISO 10993-5 and ASTM F895-11, two descriptions, the reactivity grades of zones and morphological grades of cytotoxicity, are used to identifying the degree of cytocompatibility and the cytotoxicity is determined when the grade is greater than 2. Therefore, as shown in Table 4, c(PGS-g-Arg) films with a grafting ratio of L-arginine between 7% to 18% could be considered cytocompatible.

## 4. Conclusions

Maintaining the appropriate hydration status of wounded skin and possessing the function of antimicrobial activity are important for healing. In this study, l-arginine modified poly(glycerol sebacate) materials were prepared and their mechanical strength, water vapor transmission rate, antimicrobial function, and biocompatibility were evaluated. A wide range of physical properties were preserved by varying grafting ratio of l-arginine of c(PGS-g-Arg), and the similarity of tensile modulus between normal skin and c(PGS-g-Arg) suggested that these materials might attach on the skin without discomfort. It was proven that these crosslinked materials were as good as silicone-based commercial products with the capabilities to form a barrier and decrease water vapor evaporation; thus, these materials might have potential to prevent scar formation and maintain balanced moisture condition for wound treatment. Furthermore, the antimicrobial effect of the material against *Pseudomonas aeruginosa* and *Staphylococcus aureus* were proven effective and dependent on the grafting ratios. For the intended use of wound dressing, c(PGS-g-Arg) are verified to be biocompatible. With the elasticity analogues to skin, water barrier properties, antimicrobial and biocompatibility, the novel developed l-arginine modified poly(glycerol sebacate) could be considered a promising dressing material for application toward wound treatment.

## Figures and Tables

**Table 1:** Molecular compositions and characteristics of prepolymers and crosslinked films.

Polymer Abbreviation	L-Arginine Content (mol%)	Molecular Weight	Solid Content in Ethanol (%)	Size of Crosslinked Films (L./W./T. in mm)
		Mw/Mn/PDI		
c (PGS-g- 7% Arg)	7	3060/1809/1.7	30	105/104/0.24
			50	108/108/0.46
c (PGS-g- 13% Arg)	13	3668/2221/1.7	50	106/105/0.47
c (PGS-g- 18% Arg)	18	5001/2420/2.1	50	107/107/0.50

**Table 2:** Mechanical properties of c(PGS-g-Arg) flims (n = 5).

Polymer Abbreviation	L-Arginine Content (mol%)	Mechanical Properties		
		Young’s Modulus (MPa)	UTS (MPa)	Elongation (%)
c (PGS-g- 7% Arg)	7%	1.79 ± 0.15	0.84 ± 0.08	84 ± 12
c (PGS-g- 13% Arg)	13%	0.89 ± 0.06	0.59 ± 0.08	112 ± 11
c (PGS-g- 18% Arg)	18%	0.37 ± 0.05	0.23 ± 0.02	129 ± 14

**Table 3:** Comparison of the water vapor transmission rates (WVTRs) of c(PGS-g-Arg) with commercial products by vial method (*n* = 3).

Tested Sample	Thickness (mm)	Water Vapor Transmission Rate (g/m^2^/h)
c (PGS-g- 7% Arg)	0.46 ± 0.05	6.1 ± 0.3
	0.24 ± 0.01	10.3 ± 0.5
c (PGS-g- 13% Arg)	0.47 ± 0.04	7.7 ± 0.3
c (PGS-g- 18% Arg)	0.50 ± 0.06	8.3 ± 0.4
Cica-Care ^®^	1.00	7.2 ± 1.0
SavDerm ^®^	0.50	7.8 ± 0.7
Rystora ^®^	0.30	12.2 ± 0.3
0% occlusive control of water	0.00	174.1 ± 8.2

**Table 4:** Evaluation of cytocompatibility of c(PGS-g-Arg) films (*n* = 3).

Tested Sample (*n* = 3)	Reactivity Grades of Zone	Morphological Grades of Cytotoxicity
Positive control	3, 3, 3	3, 3, 3 (54%, 50%, 48%)
Negative control	0, 0, 0	0, 0, 0 (0%, 0%, 0%)
c (PGS-g- 7% Arg)	1, 1, 1	1, 1, 1 (1%, 2%, 4%)
c (PGS-g- 13% Arg)	1, 1, 1	1, 1, 1 (4%, 2%, 7%)
c (PGS-g- 18% Arg)	1, 1, 1	1, 1, 1 (8%, 5%, 5%)
